# Early Elevation of Serum MMP-3 and MMP-12 Predicts Protection from World Trade Center-Lung Injury in New York City Firefighters: A Nested Case-Control Study

**DOI:** 10.1371/journal.pone.0076099

**Published:** 2013-10-16

**Authors:** Sophia Kwon, Michael D. Weiden, Ghislaine C. Echevarria, Ashley L. Comfort, Bushra Naveed, David J. Prezant, William N. Rom, Anna Nolan

**Affiliations:** 1 Division of Pulmonary, Critical Care and Sleep, New York University, School of Medicine, New York, New York, United States of America; 2 New York University, School of Medicine, Department of Environmental Medicine, Tuxedo Park, New York, United States of America; 3 Bureau of Health Services and Office of Medical Affairs, Fire Department of New York, Brooklyn, New York, United States of America; 4 Pulmonary Medicine Division, Department of Medicine, Montefiore Medical Center and Albert Einstein College of Medicine, Bronx, New York, United States of America,; 5 División de Anestesiología, Escuela de Medicina, Pontificia Universidad Católica de Chile, Santiago, Chile; The Ohio State University, United States of America

## Abstract

**Objective:**

After 9/11/2001, some Fire Department of New York (FDNY) workers had excessive lung function decline. We hypothesized that early serum matrix metalloproteinases (MMP) expression predicts World Trade Center-Lung Injury (WTC-LI) years later.

**Methods:**

This is a nested case-control analysis of never-smoking male firefighters with normal pre-exposure Forced Expiratory Volume in one second (FEV_1_) who had serum drawn up to 155 days post 9/11/2001. Serum MMP-1, 2,3,7,8, 9, 12 and 13 were measured. Cases of WTC-LI (N = 70) were defined as having an FEV1 one standard deviation below the mean (FEV_1_≤77%) at subspecialty pulmonary evaluation (SPE) which was performed 32 months (IQR 21–53) post-9/11. Controls (N = 123) were randomly selected. We modeled MMP's ability as a predictor of cases status with logistic regression adjusted for time to blood draw, exposure intensity, weight gain and pre-9/11 FEV_1_.

**Results:**

Each log-increase in MMP-3 and MMP-12 showed reduced odds of developing WTC-LI by 73% and 54% respectively. MMP-3 and MMP-12 consistently clustered together in cases, controls, and the cohort. Increasing time to blood draw significantly and independently increased the risk of WTC-LI.

**Conclusions:**

Elevated serum levels of MMP-3 and MMP-12 reduce the risk of developing WTC-LI. At any level of MMP-3 or 12, increased time to blood draw is associated with a diminished protective effect.

## Introduction

During the events of September 11^th^, 2001, the collapse of the World Trade Center (WTC) pulverized much of the building's glass and metal structure and released an estimated 10 million tons of particulate matter (WTC-PM).[1]–[4] The bulk particulates were composed of cement, carbon, cellulose, and several fiber types including mineral wool, fiberglass and asbestos.[3], [4] WTC-PM was collected, sieved, aerosolized, and was found to range in size from PM2.5 to PM53.[4] The elemental analysis showed that WTC-PM was composed of high levels of calcium and sulfur (mostly in the form of sulfate) and it was found to be highly alkaline; pH 9–11.[4] Furthermore, induced sputum of firefighters up to 9 months after the event contained PM2.5–53.[5] Finally, biomass exposure is a major cause of lung function loss worldwide. In addition military cohorts exposed to products of combustion and PM due to close proximity to burn pits provide further insight into the negative health effects PM exposure. [6]

Approximately 92% of the exposed rescue workers were subsequently enrolled in the Fire Department of New York-World Trade Center-Medical Monitoring and Treatment Program.[7] Serum samples and spirometry were obtained within six months of exposure at medical monitoring entry (MME). Despite high levels of exposure the FDNY rescue worker cohort had a very heterogeneous response in regards to their lung function.[8]–[10] Most stabilized, a subset of rescue workers recovered lung function while others progressed and their FEV1 declined to less than their lower limit of normal (LLN). We have previously defined the persistent loss of FEV1 to less than LLN in this FDNY exposed population as World Trade Center Lung Injury (WTC-LI).[10]–[13] Workers with this progressive disease had bronchial wall thickening and air trapping on computed tomography (CT), suggesting airway injury as the predominant lung pathology.[14] Lower Manhattan residents with WTC exposure have similar findings.[8], [12], [14]–[21] This suggests that airway disease is the most common manifestation of WTC-LI.

We have previously investigated serum inflammatory, metabolic syndrome and cardiovascular biomarkers and found them to be predictors of FEV_1_ decline and WTC-LI.[11], [13], [22], [23] More specifically, we found that myeloperoxidase (MPO) and soluble vascular cell adhesion molecule (sVCAM) can predict recovery from the original injury.[24] These results demonstrate that patho-physiological processes in the lung caused by dust and smoke exposure are reflected by biologically active protein expression in serum.

The role of proteases has been studied in the setting of many diseases including cancer, coronary disease, chronic obstructive pulmonary disease (COPD), and cigarette-induced chronic lung diseases.[25]–[29] Increased protease activity is a component of many diseases, including cigarette-induced chronic lung disease and other causes of accelerated lung function decline.[25]–[28] Of the known proteases, matrix metalloproteinases (MMPs) have been of particular interest in their role in lung remodeling, and have been found to be central to the pathogenesis of airway disease such as COPD. MMPs are a family of Zn^2+^-dependent proteases that can catabolize and degrade the extracellular matrix. Many are known intermediates of each other, and their levels are affected by environmental factors such as hypoxia, inflammation and oxidative stress. The utility of MMPs as biomarkers of lung disease severity and prognostic indicators are of key interest in many studies.[30] Genetic association studies with MMPs demonstrate a strong association with lung disease.[25], [31], [32] MMP-9 has been studied in induced sputum of WTC-dust-exposed firefighters, and was found to be related to the time spent during recovery efforts at the WTC site.[5] MMP-9 has also been used to predict lung function decline in the setting of α1-antitrypsin deficiency.[33] However, the overall importance of the proteases in the development and modulation of lung disease needs further study.[34]–[37]

This study investigates the levels of MMPs present in serum after exposure to WTC dust in rescue workers as potential systemic biomarkers predicting susceptibility to WTC-LI. It tests the hypothesis that proteases present within three months of 9/11/2001 can predict airway injury years later.

## Methods

### Study participants

This study was approved by both Montefiore Medical Center and New York University IRB. All subjects signed informed Institutional Review Board-approved consent at the time of enrollment allowing analysis of their information and samples for research (Montefiore Medical Center; #07-09-320 and New York University; #11-00439). De-identified data devoid of sensitive information acquired through WTC National Institute of Occupational Safety and Health (NIOSH) funding is available to health researchers and others in accordance with the Zadroga Act and in full compliance with the Centers for Disease Control and Prevention (CDC) and the Agency for Toxic Substances and Disease Registry (ATSDR) Policy on Releasing and Sharing Data by request to the WTC Health Program directors.

Prior to 9/11, all subjects had spirometry as part of an annual physical. At medical monitoring entry (MME), serum samples and the first post-9/11 spirometry were obtained (Portascreen Spirometry; S&M Instruments). The population was evaluated and monitored, and N = 13,234 symptomatic firefighters were referred to subspecialty pulmonary evaluation (SPE) if they became symptomatic.[10] Between 10/1/2001 and 3/10/2008, N = 1720/13,234 presented to a single center for SPE that included methacholine challenge, diffusing capacity of the lung for carbon monoxide (DL_CO_), bronchodilator response (Jaeger Masterscreen, Viasys Healthcare) and chest CT. Exposure intensity was defined as high if firefighters reported arrival to the site the morning of 9/11, and intermediate if they arrived in the afternoon of 9/11 or on 9/12.

### Study design

Similar to previous studies a nested case-control design was used to measure the association of serum analytes with case status. [11], [13], [23], [38] Utilizing a cohort control is logistically efficient and cost-effective for biomarker studies because it samples a control population that approximates the larger cohort.[11], [13], [23], [38], [39] The study design is also advantageous because it minimizes batch-bias and freeze-thaw problems associated with biomarker discovery.[38]

Subjects were included in the study if they were never-smoking male firefighters who had reliable National Health and Nutrition Examination Survey (NHANES) normative data for predicted FEV1, post-9/11 FDNY PFTs within 200 days of 9/11, and pre-9/11 FEV1 >75% pred (n = 801 (47%) out of 1720). [10], [12], [24] Those with the most abnormal FEV1% at SPE were identified as cases of WTC-LI. Cases (n = 100) of WTC-LI were defined as being within one standard deviation of the lowest FEV1% pred of the cohort at the time of SPE. The cohort control (N = 171) was randomly selected from the baseline cohort after stratification on BMI and FEV_1_ at MME. Controls (n = 153/171) met inclusion criteria. Serum was available for the final cohort of n = 123/153 cohort controls and n = 70/100 cases.

### Serum biomarker assays

Blood drawn at MME was allowed to stand for 1 hour at room temperature, and then centrifuged at 1,800g for 10 minutes. Serum was stored at −80°C (Bio-Reference Laboratories, Inc. Elmwood Park, NJ), thawed once at 4°C, and assayed using MMP panel (Procarta/Affymetrix), which had a detection range of 2.44–40,000 ng/mL. Panels were read on Luminex 200-IS (Luminex Corporation, Austin, TX) and analyzed with MasterPlexQT software (Ver. 1.2; MiraiBio, Inc., San Diego, CA). MMP values were internally validated using manufacturer controls and standards of known concentrations. Each plate contained a 1∶2 ratio of cases to controls to control for batch artifact.

### Statistical analysis

We tested normality using the Shapiro-Wilk test and Q-Q plots. We used Mann–Whitney U test for between group comparisons, as appropriate. Pearson's Chi-squared test was used for inferences on proportions.

Odds ratios were calculated by multivariable logistic regression using case definition as the outcome variable. Since biomarker levels were skewed, values were log-transformed and included as continuous covariates in the model. The Hosmer-Lemeshow goodness-of-fit test was used to assess the calibration of the model. The model discrimination was quantified using the receiver operating characteristic (ROC) area under the curve (AUC).

Hierarchical clustering was performed using Cluster 3.0 (Ver. 1.47, Michael Eisen: Stanford University; Michiel de Hoon: University of Tokyo) and Java Treeview (Ver.1.1, Saldanha).[40], [41] Data was adjusted by log-transformation and centered on the median.MMP serum levels were clustered by average linkage using Spearman's Rank Correlation as the similarity metric.

Data are expressed as median (interquartile range, IQR) or Odds Ratio (95% confidence interval), unless otherwise stated. A two sided p value less than 0.05 was considered significant. Database management and analyses were performed using SPSS 20 (IBM, Armonk, NY) and STATA/SE 12 (StataCorp, College Station, TX).

## Results

### Participants

**Figure 1:**
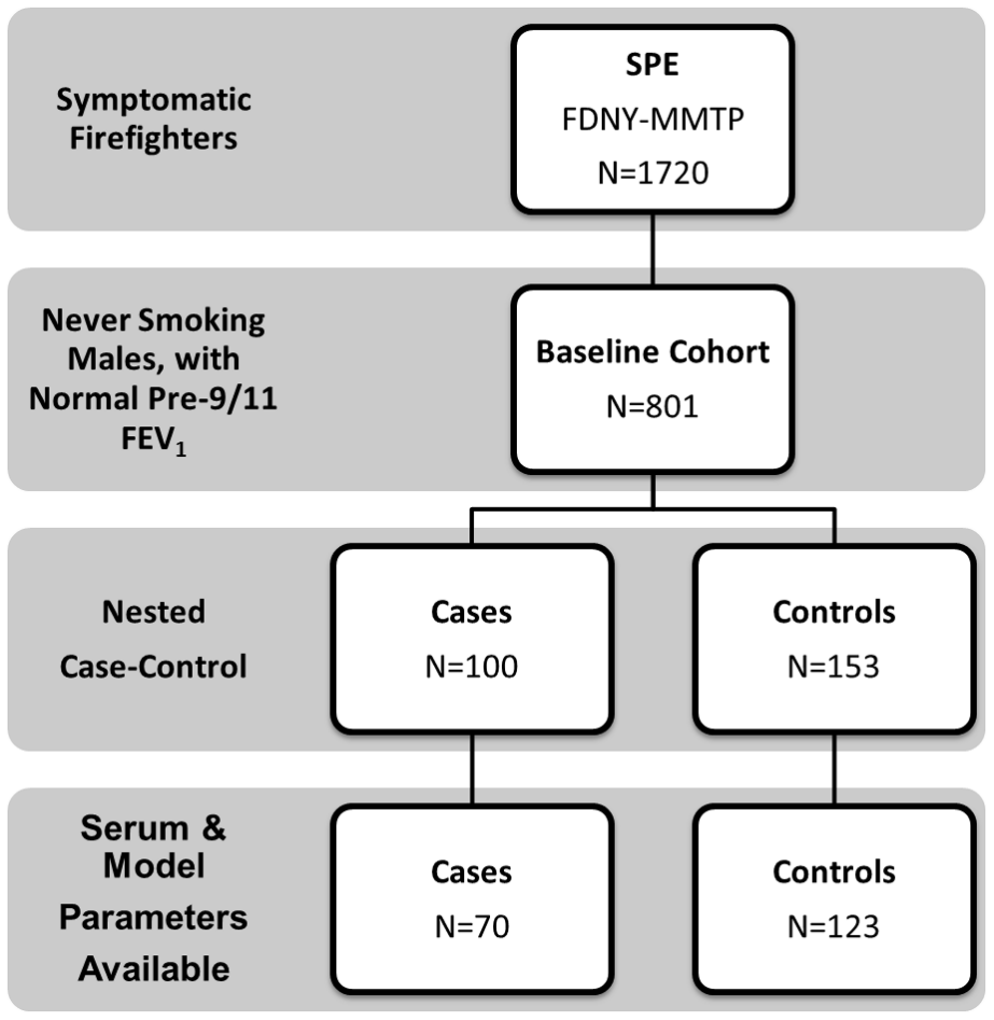
Study Design. Derivation of Study Cohort from N = 1720 symptomatic FDNY firefighters who presented for subspecialty pulmonary testing. Serum available and inclusion criteria met for N = 70/100 cases and N = 123/153 controls.

Derivation of the baseline cohort, cases and controls are described in Figure 1. N = 123 controls were compared N = 70/100 cases. Cases and controls were not significantly different in exposure intensity, time to MME or SPE, BMI at MME, years of service, or age [Table 1]. However, BMI at SPE and change in BMI between MME and SPE showed that cases had significantly increased BMI compared to controls at the later time point. Median time between 9/11/2001 and SPE for the baseline cohort was 34 months (IQR range for all groups 25–57).There was no significant difference in time to MME or SPE for controls and cases [Table 1].

**Table 1:** Demographics of Cohort.

	Date/Event	Baseline Cohort	Susceptible Cases	Sub-Cohort Controls	p
N		801	70	123	
**WTC Exposure, N(%)**	High	197(25%)	18(26%)	21(17%)	0.151
	Intermediate	604(75.4%)	52(74%)	102(83%)	
**9/11 to PFT, Months** *	MME	2.7(2–4)	2.7(2–4)	2.5(2–3)	0.145
	SPE	33.8(25–57)	32.6(21–53)	35.5(26–55)	0.327
**BMI, kg/m^2^** *	MME	28.0(26–30)	29.0(27–31)	28.0(26–31)	0.106
	SPE	28.9(27–31)	29.6(27–34)	29.0(27–31)	0.018
	Change	0.8(–0.1–1.8)	1.1(0–2.3)	0.6(–0.4–1.7)	0.003
**Years of Service** *	9/11/01	13(7–19)	15(8–18)	14(7–18)	0.907
**Age** *	9/11/01	40(36–45)	40(36–45)	42(37–46)	0.764

* Median (IQR).

WTC, World Trade Center; MME, Medical Monitoring Exam; SPE, Subspecialty Pulmonary Exam; PFT, Pulmonary Function Test; BMI, Body Mass Index.

### Longitudinal lung function in cases and controls

**Figure 2:**
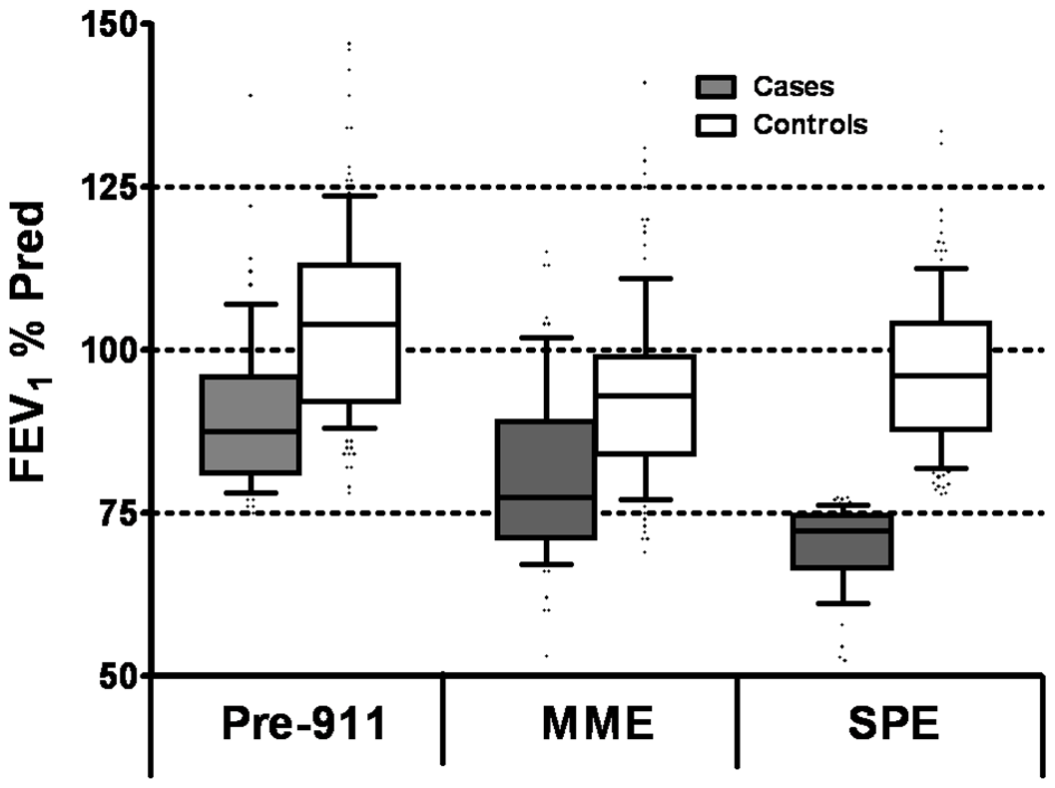
FEV_1_% Predicted based on NHANES of Serial PFTs of Cases and Controls. Median and IQR are represented by box plots, with median drawn in the middle of the box. The extremes of the error bars (whiskers) represent 10–90% percentile.

Three sequential pulmonary functions tests (PFT) were performed on this cohort; prior to exposure (Pre-9/11), at MME, and at SPE [Figure 2] [Table 2].

**Table 2:** Longitudinal Lung Function Assessment of Cohort.

Time	Variable	Cases	N	Controls	N	p
**Pre-9/11** *	**FEV_1%_**	88(81–96)	70	104(92–113)	123	<0.001
	**FEV_1_/FVC**	81.7(78–86)	70	84.9(81–88)	123	0.001
**MME** *	**FEV_1%_**	78(71–89)	70	93(84–99)	123	<0.001
	**FEV_1_/FVC**	81.4(76–86)	70	83.8(80–87)	123	0.016
**SPE**	**FEV_1%_** *	72(66–75)	70	96(88–104)	123	<0.001
	**FEV_1_/FVC** *	71.1(65–77)	70	77.1(73–81)	123	<0.001
	**BDR** * **%**	15(7–29)	46	5(2–8)	46	<0.001
	**BDR**≥**12%, (N,%)**	30(65)	46	10(22)	46	<0.001
	**MCT slope** *	0.24(0.06–1.78)	34	0.05(0.03–0.11)	102	0.001
	**PC_20_**≤**10 mg/mL, (N,%)**	17(50)	34	19(19)	102	<0.001
	**TLC%** *	96(83–106)	47	103(98–109)	53	0.002
	**RV,% Pred** *	130(109–157)	47	123(111–140)	53	0.525
	**DL_CO_%** *	96(85–107)	46	107(101–116)	52	<0.001
	**VA%** *	83(75–89)	35	94(87–101)	43	<0.001
	**DL_CO_/VA** *	122(113–134)	36	117(105–122)	40	0.038
	**Nodule, (N,%)**	18(45)	40	25(39)	64	0.550
	**Air Trapping, (N,%)**	22(55)	40	27(42)	64	0.203
	**BWT, (N,%)**	13(33)	40	23(36)	64	0.833

* Median (IQR).

FEV_1_, Forced Expiratory Volume in one second; FVC, Forced Vital capacity; BDR, Bronchodilator Response; MCT, Methacholine Challenge Testing; PC_20_, Provocative concentration of methacholine that results in a 20% drop in FEV_1_; TLC, Total Lung Capacity; RV, Residual Volume; DL_co,_ Diffusing Capacity of the Lung for Carbon Monoxide; VA, Alveolar Ventilation; BWT, Bronchial Wall Thickening.

Although cases had lower pre-9/11 FEV_1_ (88%) than the controls (104%), all FDNY workers had normal FEV_1_ by design. The controls and cases suffered similar decline of FEV_1_ in the interval from pre-9/11 to MME (11% for the controls and cases). Importantly, controls regained some FEV_1_ between MME and SPE, whereas cases continued to lose lung function over this interval.

Cases had more decline in FEV_1_/FVC ratio between MME and SPE (0.81 to 0.71) than controls (0.84 to 0.77) [Table 2]. Evidence of airway reactivitytracked with the magnitude of changes in FEV_1_/FVC ratio. The median bronchodilator response in cases was 15%, while FEV_1_ increased only 5% in controls (p<0.001). Using a 12% FEV_1_ increase post-bronchodilator as a threshold, 65% of the cases had bronchodilator response compared to only 22% in controls. Similarly, the methacholine slope was at least 4-fold higher in cases compared to controls. Using a 20% decrease in FEV_1_ at the 10 mg/ml methacholine dose as a threshold (PC_20_≤10 mg/mL, a slope of 0.3125% FEV_1_ decline/mg of methacholine), 50% of the cases had airway reactivity compared to 19% in controls. There was no difference in RV% Predicted. Cases and controls were significantly different in Total lung capacity, DL_CO_, alveolar ventilation (VA) and DL_CO_/VA [Table 2]. We used CT imaging to investigate if cases and controls had different radiographic measures of lung injury. There was no difference in nodularity, air trapping, or bronchial wall thickening. Interestingly, the cohort had high prevalence of each of these radiographic abnormalities.

### Serum MMPs

MMP-1,3,8 and 12 were significantly lower in cases that would progress to WTC-LI than controls who would subsequently recover some of the lung function lost after WTC exposure. There was no difference in MMP-2,7,9 and 13 expression in cases and control [Table 3].

**Table 3:** Serum Biomarkers.

Analyte	Cases	Controls	p
pg/mL	N = 70	N = 123	
**MMP-1** *	387(116–864)	775(296–1368)	0.001
**MMP-2**	2840(1281–5130)	3020(1815–4640)	0.268
**MMP-3** *	3194(1962–7542)	7653(3320–13765)	<0.001
**MMP-7**	293(67–396)	222(96–320)	0.382
**MMP-8** *	2(2–20)	2(2–174)	0.030
**MMP-9**	25610(12222–74000)	23490(11196–47084)	0.548
**MMP-12** *	35(7–218)	66(22–313)	0.008
**MMP-13**	58(9–109)	75(3–141)	0.355

* p<0.05; All values shown as median (IQR).

**Figure 3:**
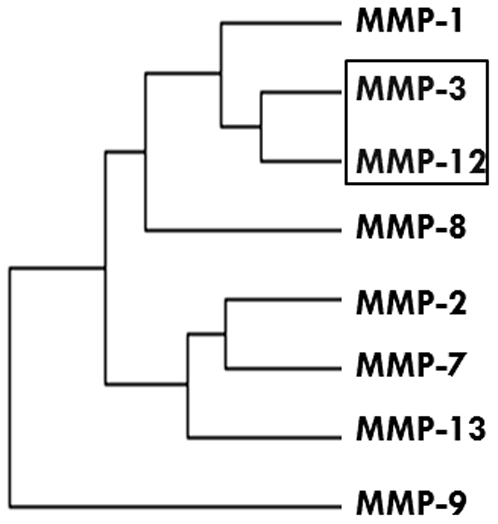
Hierarchical Clustering of Serum MMPs. Clustering of MMPs in the cohort, N = 193 showed that MMP3 and MMP-12 clustered together.

Hierarchical clustering of serum biomarkers tested coordinate expression of the MMPs [Figure 3]. Clustering the MMPs in the entire study cohort formed two distinct clusters. The MMPs (1, 3, 8, 12) that were significantly lower in the cases when compared to controls clustered together. MMP-3 and MMP-12 clustered in the entire study cohort [Figure 3] and in the cases and controls when there MMPs were clustered separately, data not shown.

### Predictive risk models

We then tested if the MMPs that were different between cases and controls predicted future lung function with logistic regression. All MMP levels remained as continuous variables and were log-transformed to approximate a normal distribution [Table 4].[38] Covariates in the adjusted models are days to blood draw (9/11 to MME), change in BMI between blood draw and case definition (MME to SPE), WTC exposure intensity, and pre-9/11 FEV_1_% predicted.

**Table 4:** Models of Susceptibility to Lung Injury.*

Model	OR (95% CI)	AUC	HL
**MMP-1**	0.476 (0.261–0.866)	0.819 (0.755–0.882)	0.034
**Time to Serum**	1.013 (1.000–1.027)		
**MMP-3**	0.267 (0.121–0.589)	0.832 (0.771–0.893)	0.182
**Time to Serum**	1.014 (1.000–1.027)		
**MMP-8**	0.675 (0.410–1.111)	0.810 (0.745–0.874)	0.075
**Time to Serum**	1.010 (0.997–1.024)		
**MMP-12**	0.462 (0.260–0.821)	0.818 (0.755–0.881)	0.506
**Time to Serum**	1.013(1.000–1.027)		

* Each Model Includes: ΔBMI between MME and SPE, Exposure Group, Pre-9/11 FEV_1_% Predicted, Time to MME (days) and MMP (Log_10 _pg/mL).

OR, Odds Ratio; CI, Confidence Interval; AUC, Area Under the Curve; HL, Hosmer Lemeshow.

Four models were developed using case definition as the outcome variable, and assessed for quality using ROC analysis and Hosmer-Lemeshow goodness of fit statistic. MMP-8 was not a significant predictor of lung function after adjustments. Although MMP-1, MMP-3, and MMP-12 demonstrated significant predictive ability, MMP-1 was discounted from further analysis because it failed Hosmer-Lemeshow goodness of fit test. Time to serum draw was also a significant covariate for MMP-3 and MMP-12; every day post-exposure increased the odds of developing WTC-LI by 1.4% and 1.3% respectively. The odds ratios for MMP-3 and MMP-12 were 0.267 and 0.462 respectively, indicating a protective function of elevated levels of the proteases. MMP-3 and MMP-12 demonstrated excellent predictive ability with AUC of 0.832 and 0.818 respectively. The MMP-12 and MMP-3 models have a sensitivity of 58.6% and specificity of 86.2%.

### The effect of time to blood draw and MMP level of the probability of developing WTC-LI

**Figure 4:**
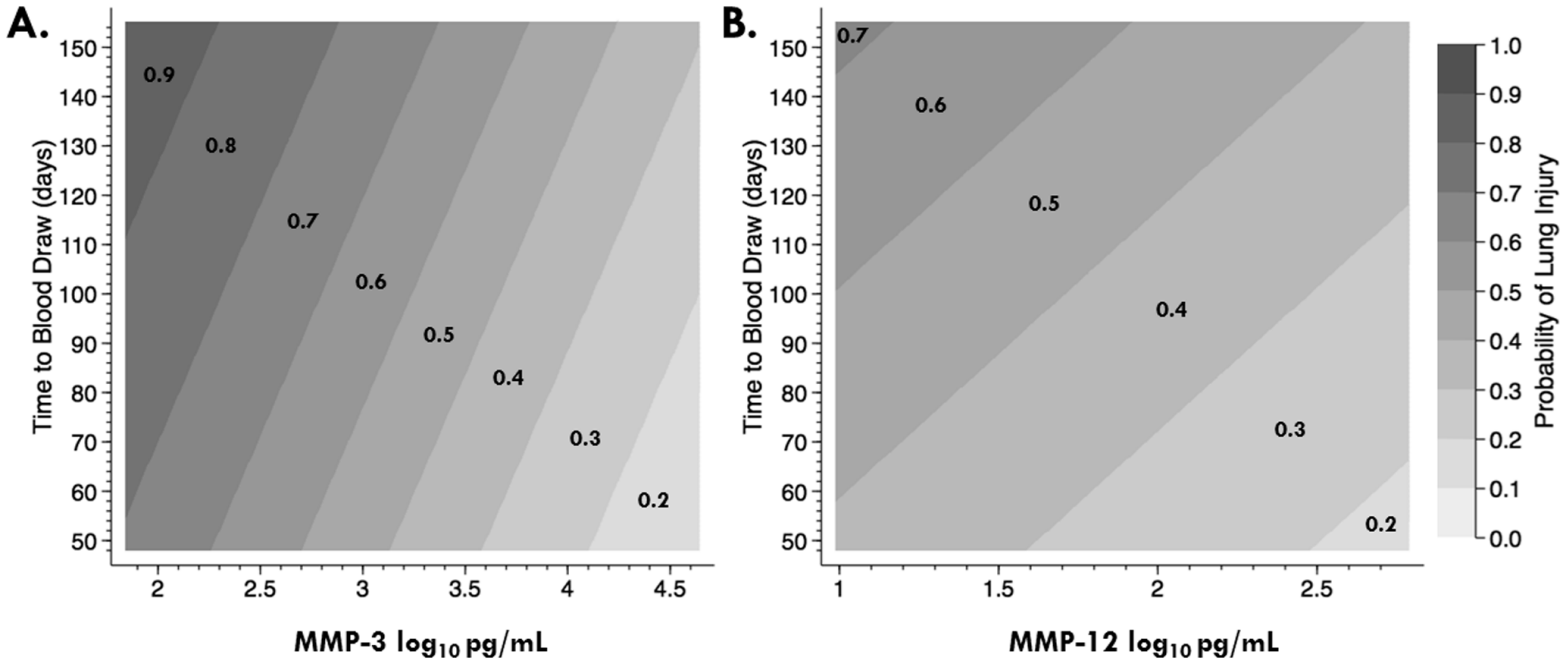
Probability of Developing WTC-LI. Contour Plots express probability isopleths for the development of WTC-LI with all other covariates held constant. When either MMP-3 (A) or MMP-12 (B) increases, the probability of lung injury decreases. As time to blood draw increases, the probability of lung Injury increases.

To better understand the effect of time on the association between serum MMP level and WTC-LI, we utilized a contour plot [Figure 4]. The chance of developing WTC-LI is shown as a probability isopleth. MMP-3 and MMP-12 both show with increasing serum levels, probability of WTC-LI is decreased. Conversely, increasing time to blood draw increased the probability of WTC-LI. Specifically, as the level of MMP-3 increased from 2-4.5 Log_10_ pg/mL, the probability of WTC-LI decreased from 90% to 20% [Figure 4A]. As the serum level of MMP-12 increased from 1–2.5 Log_10_ pg/mL, the probability of developing WTC-LI ranged from 70% to 20% respectively [Figure 4B]. As time to blood draw increased, the probability of developing WTC-LI ranged from 70–90% in the MMP-3 model and from 40–70% for the MMP-12 model.

## Discussion

We report that early elevated expression of MMP-3 and MMP-12 in serum within 200 days after WTC exposure predicts protected lung function over the subsequent seven years (between 2001–2008). We found that MMP-3 and MMP-12 levels of the cohort were comparable to other published patient populations, including healthy controls and patient cohorts with emphysema or rheumatoid arthritis.[30], [42] MMP-3 was found to be more protective than MMP-12 with an odds ratio of 0.267 compared to 0.462. Both biomarker models displayed robust predictive ability by logistic regression with AUC>0.8. Presenting later to MME and blood draw predicted higher probability of developing WTC-LI. As time to blood draw increased, the probability of developing WTC-LI ranged from 70–90% in the MMP-3 model and from 40–70% for the MMP-12 model.

This cohort provides a unique opportunity identify biomarkers of susceptibility to dust induced lung injury. First, FEV_1_ was measured prior to exposure. Second, after the WTC collapse produced a massive acute dust exposure, serum was drawn early during the evolution process to disease. Longitudinal FEV_1_ measurements allowed us to define case status years after the serum was drawn. The cohort is also large enough to study only never-smokers, eliminating a major confounder of reduced FEV_1_. The development of FEV_1_ loss in obstructive lung disease is a multifactorial process. MMPs are central in the disease pathogenesis of obstructive lung disease, and are thus of interest in this population. MMPs are involved in parenchymal destruction and lung remodeling, and their quantification allows us to start to understand their role in the development of FEV_1_ loss in this WTC exposed population. Furthermore, they are involved in separate pathways of disease attenuation than those of inflammation, cardiovascular disease, and metabolic syndrome that were the focus of our prior work.

Cases and controls differed in several ways that impacted future analyses. Incident cases gained significantly more weight than controls by the time of SPE. BMI is a known cause of decreased lung function, and is thus a possible confounder. Because our case definition of WTC-LI relies on FEV_1_ at SPE, it is heavily impacted by pre-9/11 FEV_1_ and BMI at SPE, which are both significantly different between cases and controls prior to model building. For this reason, we adjust for pre-9/11 FEV_1_ and change in BMI (as a measure of weight gain) as confounders in the logistic regression model. The change in BMI in the logistic regression is simply used as a marker of weight gain. The model remains robust whether the weight gain is measured as change in BMI or BMI at SPE.

Cases and controls suffered similar acute reductions in FEV_1_ from pre-9/11 to MME. This is consistent with the similar WTC exposure intensities in both case definitions. However, controls had higher pre-9/11 FEV_1_ than susceptible cases (102 vs 88%). The differences in pre-9/11 FEV_1_ could reflect predisposition to lung injury after non-tobacco smoke exposure during the population's 18 years of service in fighting fires. This is suggested by continued deterioration of cases in FEV_1_ compared to the relative recovery of controls years after the acute WTC exposure. Because cases were defined by a threshold of FEV_1_ of <77% at SPE, those with pre-9/11 FEV_1_ close to threshold values were more likely to meet case definition. Cases also had markedly diminished airway function across multiple measures after exposure, including FEV_1_, lower FEV_1_/FVC ratio, more bronchodilator response, and steeper methacholine response slope.

We observe that increased MMP-3 and 12 expression reduces the odds of subsequent lung injury. Also, the time to medical monitoring enrollment and serum draw post-exposure was an independent predictor of lung injury. Those who had serum obtained two months post-9/11 (early presenters) had lower risk of lung injury than those who had serum obtained four months post-9/11 (late presenters). This result could be due to selection bias with individuals entering medical monitoring later at a more advanced stage of disease. Alternately, delay in early interventional treatment for those who enrolled in medical monitoring later may have inhibited their ability to recover from the exposure. We investigated this possibility, but did not find differences between early and late presenters in known confounders such as exposure intensity or pre-9/11 FEV_1_. There was a significant increase in BMI in the late presenters, but the effect of time did not change with BMI as a covariate in the logistic models. Time to serum draw was not a significant covariate with all other serum biomarkers of risk or protection that we have reported.[11], [13], [23] This suggests that the predictive character of MMP expression over time is distinct from other biomarkers of metabolic syndrome, vascular injury or inflammation. Another possible interpretation of the effect of time is early elevation of MMP reflects normal healing, while persistent MMP elevation occurs with non-resolving inflammation. Since we do not have serial serum samples from each individual, we are unable to determine if the association of time to serum draw post-9/11 with risk of lung injury is due to selection bias or reflects underlying biology.

The protective role of MMP-12, also known as macrophage elastase, in this cohort was surprising since most studies on patients with established lung disease demonstrate elevated MMP-12 as a biomarker of disease severity in COPD patients.[25] Nevertheless, there exists murine data for potential protective roles of MMP-12 in the setting of non-small cell lung cancer.[43] The data is consistent with the hypothesis that after injury, elevated MMP-3 and MMP-12 reduce odds of developing WTC-LI. In this study, MMP-12 was assayed soon after injury, during the period of disease evolution, but years before disease was diagnosed. This makes reverse causation unlikely. It is possible that when irreversible pulmonary disease is established, increased MMP-12 is a consequence of disease severity and not its cause. Thus, MMPs would be elevated in more advanced disease due to induction of an infective or counter-productive repair response.

This is the first paper that describes a protective function of elevated MMP-3, stromelysin-1, in humans. This novel correlation needs to be shown in other lung injury cohorts prior to investigating the relationship of progressive injury pathways and MMP-3 expression. MMP-3 has been shown to be protective in murine models of atherosclerosis.[29] Murine models of intestinal sepsis also showed that deficiency in MMP-3 delayed clearance of bacteria and impaired migration of CD4^+^T-cells.[29]

This nested case control study has several limitations. The cohort was narrowly defined, reducing generalizability of these results. Specifically, the vast majority of FDNY workers were exposed at the time of the WTC destruction. The few unexposed workers were markedly less healthy than the exposed workers pre-9/11 and most were not on active duty at the time of the event. Therefore, all the study participants were highly exposed to WTC dust and products of combustion. Since there is no unexposed control group with comparable lung function, years of service, age, racial background and serum available it is therefore not feasible to construct an unexposed control. Also, using FEV_1_ to define case status may produce misclassification of disease. However, the authors maintain that using FEV_1_ is the simplest, most robust, and best validated single measure of airway injury in an obstructed cohort. Since the design reduced the potential for selection bias, misclassification of disease is non-differential. The reported associations therefore likely underestimate the true strength of association between the serum biomarkers and subsequent FEV_1_. Although the serum biomarkers are expressed years before the disease is defined, the results are correlations and do not imply causation. This restricts our ability to assess the impact of WTC exposure to the observed biomarker disease relationship. Biomarker associations with lung injury must also be investigated in independent cohorts.

WTC exposure impacted multiple distinct injury and repair pathways. One interpretation of the findings is that biomarkers that reduce the risk of WTC-LI reflect effective repair after an acute dust induced lung injury. We report that elevated MMP-3 and MMP-12 are associated with a reduction in the odds of developing WTC-LI years later. Finally, we show that time to serum sample acquisition is independently associated with the probability of lung injury and highlights the utility of serum sampling efforts continuing for some time. We will investigate in future studies about the relationship between genetic polymorphisms that may improve or change the predictive ability of MMP-3 and MMP-12. Also, we will analyze longitudinal samples of serum on the same cohort to understand how chronicity of the biomarkers impact disease development.
